# *In Vitro* Schistosomicidal Activity of Some Brazilian Cerrado Species and Their Isolated Compounds

**DOI:** 10.1155/2012/173614

**Published:** 2012-08-08

**Authors:** Nayanne Larissa Cunha, Camila Jacintho de Mendonça Uchôa, Lucas Silva Cintra, Herbert Cristian de Souza, Juliana Andrade Peixoto, Claudia Peres Silva, Lizandra Guidi Magalhães, Valéria Maria Meleiro Gimenez, Milton Groppo, Vanderlei Rodrigues, Ademar Alves da Silva Filho, Márcio Luís Andrade e Silva, Wilson Roberto Cunha, Patrícia Mendonça Pauletti, Ana Helena Januário

**Affiliations:** ^1^Universidade de Franca, Avenida. Dr. Armando Salles de Oliveira 201, 14404-600 Franca, SP, Brazil; ^2^Centro Universitário Claretiano, Rua Dom Bosco 466, 14300-000 Batatais, SP, Brazil; ^3^Faculdade de Filosofia, Ciências e Letras, Universidade de São Paulo, Avenida Bandeirantes 3900, 14040-901 Ribeirão Preto, SP, Brazil; ^4^Faculdade de Medicina de Ribeirão Preto, Universidade de São Paulo, Avenida Bandeirantes 3900, 14049-900 Ribeirão Preto, SP, Brazil; ^5^Departamento Farmacêutico, Universidade Federal de Juiz de Fora, Rua José Lourenço Kelmer, s/no, 36036-900 Juiz de Fora, MG, Brazil

## Abstract

*Miconia langsdorffii* Cogn. (Melastomataceae), *Roupala montana* Aubl. (Proteaceae), *Struthanthus syringifolius* (Mart.) (Loranthaceae), and *Schefflera vinosa* (Cham. & Schltdl.) Frodin (Araliaceae) are plant species from the Brazilian Cerrado whose schistosomicidal potential has not yet been described. The crude extracts, fractions, the triterpenes betulin, oleanolic acid, ursolic acid and the flavonoids quercetin 3-*O*-**β**-D-rhamnoside, quercetin 3-*O*-**β**-D-glucoside, quercetin 3-*O*-**β**-D-glucopyranosyl-(1-2)-**α**-L-rhamnopyranoside and isorhamnetin 3-*O*-**β**-D-glucopyranosyl-(1-2)-**α**-L-rhamnopyranoside were evaluated *in vitro* against *Schistosoma mansoni* adult worms and the bioactive *n*-hexane fractions of the mentioned species were also analyzed by GC-MS. Betulin was able to cause worm death percentage values of 25% after 120 h (at 100 **μ**M), and 25% and 50% after 24 and 120 h (at 200 **μ**M), respectively; besides the flavonoid quercetin 3-*O*-**β**-D-rhamnoside promoted 25% of death of the parasites at 100 **μ**M. Farther the flavonoids quercetin 3-*O*-**β**-D-glucoside and quercetin 3-*O*-**β**-D-rhamnoside at 100 **μ**M exhibited significantly reduction in motor activity, 75% and 87.5%, respectively. Biological results indicated that crude extracts of *R. montana*, *S. vinosa*, and *M. langsdorffii* and some *n*-hexane and EtOAc fractions of this species were able to induce worm death to some extent. The results suggest that lupane-type triterpenes and flavonoid monoglycosides should be considered for further antiparasites studies.

## 1. Introduction

Schistosomiasis, caused by trematode flatworms of the genus *Schistosoma*, is one of the most significant, neglected tropical diseases in the world. This disease still displays significant values of prevalence and morbidity, affecting more than 200 million people worldwide and resulting in as many as 280,000 deaths each year with over 779 million people at risk of infection, despite the great advances in treatment and prevention [1–3]. Praziquantel (PZQ) and oxamniquine are the drugs that are currently available for the treatment of schistosomiasis. However, low cure rates and treatment failure following PZQ administration have been reported in patients [4]. The Brazilian savanna, known as Cerrado, comprises a very rich and characteristic flora that covers more than 2 million square kilometers of Brazilian inland. It is a biome that is even more threatened than the Amazon rainforest [5, 6]. Cerrado is the world's most biodiverse savanna, with high degree of endemism and very high rate of environmental loss, thus regarded as a biodiversity hotspot [7]. This large biodiversity puts the country in a strategic position for the development of rational and sustained exploration of new metabolites of therapeutic value [8]. The use of online techniques has assisted in the rapid identification of active compounds in Cerrado species. As an example we can cite the online identification of chlorogenic acids, sesquiterpene lactones, and flavonoids in the Brazilian Cerrado species *Lychnophora ericoides * by HPLC-DAD-MS and HPLC-DAD-MS/MS [9]. Moreover, the literature lacks reports on the chemical composition of the selected species, namely *Roupala montana *Aubl. (Proteaceae), *Struthanthus syringifolius* (Mart.) (Loranthaceae), and *Schefflera vinosa* (Cham. & Schltdl.) Frodin (Araliaceae). Nevertheless, isolation of triterpenes, saponins, and caffeoylquinic acid derivatives from *Schefflera* and of terpenes, lignans, flavonoids, carbohydrates, fatty acids, amides, phenylpropanoids, tannins, and alkaloids from *Struthanthus* has been reported [10–14]. *Miconia langsdorffii *Cogn. (Melastomataceae) has been described to display antileishmanial activity, and isolation of the triterpenes ursolic acid and oleanolic acid from this plant species has been reported [15]. As part of our continuing interest in Brazilian Cerrado species with a view to finding out schistosomicidal agents and new drugs with action against *Schistosoma *species [16–20], we now report on the evaluation of the schistosomicidal activity of the extracts and fractions obtained from *M. langsdorffii*,* R. montana*, *S. syringifolius*, and *S. vinosa*, as well as on the activity of the isolated compounds betulin (**1**), oleanolic acid (**2**), ursolic acid (**3**), quercetin 3-*O*-*β*-d-glucoside (**4**), quercetin 3-*O*-*β*-d-glucopyranosyl-(1-2)-*α*-l-rhamnopyranoside (**5**), isorhamnetin 3-*O*-*β*-d-glucopyranosyl-(1-2)-*α*-l-rhamnopyranoside (**6**), quercetin 3-*O*-*β*-d-rhamnoside (**7**).

## 2. Material and Methods

### 2.1. General

^1^H and ^13^C NMR spectra were recorded in pyridine-*d*_5_ for triterpenes and DMSO-*d*_6_ for flavonoids using TMS as internal standard. Both analytical and preparative HPLC separation analyses were carried out on a Shimadzu LC-6AD system equipped with a degasser DGU-20A5, a UV-VIS detector SPD-20A series, a communication bus module CBM-20A, and a Reodyne manual injector. Separations of the micromolecules were accomplished on a Shimadzu Shim-pack ODS (particle diameter 5 *μ*m, 250 × 4.60 mm, and 250 × 20 mm) columns equipped with a pre-column of the same material. The MeOH used in the experiments was HPLC grade, J. T. Baker. Ultrapure water was obtained by passing redistilled water through a Direct-Q UV3 system from Millipore.

### 2.2. Plant Material

 The aerial parts of *Roupala montana* Aubl. (Proteaceae) and *Schefflera vinosa* (Cham. & Schltdl.) Frodin (Araliaceae) were collected in Luis Antonio, State of São Paulo, Brazil, in May 2008; *Miconia langsdorffii* Cogn. (Melastomataceae) was collected in Serra Azul, State of São Paulo, Brazil, in March 2009; and *Struthanthus syringifolius *(Mart.) (Loranthaceae) was collected in Itamarandiba, State of Minas Gerais, Brazil, in October 2009. The materials were identified by Prof. V. M. M. Gimenez and Prof. M. Groppo. Vouchers specimens (SPFR12166, SPFR12167 SPFR12288, and SPFR12171, resp.) were deposited in the Herbarium of Faculdade de Filosofia Ciências e Letras de Ribeirão Preto, University of São Paulo, Brazil (Herbarium SPFR).

### 2.3. Extraction and Isolation

 The aerial parts of *M. langsdorffii* (0.5 kg),* R. montana *(0.9 kg), *S. syringifolius* (1.5 kg), and *S. vinosa* (0.5 kg) were powdered and exhaustively extracted by maceration at room temperature using EtOH for the three former plants, while EtOH/H_2_O 8 : 2 (v/v) was employed for *S. vinosa*. After filtration, the solvent was removed under reduced pressure, yielding 7.8 g, 24 g, 39 g, and 31 g of crude extract from the above mentioned plants, respectively. The crude extracts of *R. montana *(**RM**, 30 g), *S. syringifolius *(**SS**, 20 g), and *S. vinosa* (**SV**, 30 g) were then dissolved in MeOH/H_2_O 2 : 8 (v/v) and successively partitioned with *n*-hexane, EtOAc, and *n*-BuOH. After solvent removal, each partition phase furnished 4.0 g, 5.9 g, 10.3 g, and 5.1 g of material for *R. montana*; 5.3 g, 2.9 g, 3.1 g, and 3.2 g for *S. syringifolius*; and 3.9 g, 7.0 g, 15.5 g, and 2.8 g for *S. vinosa*. The *n*-hexane fractions (200 mg) of each extract were chromatographed over silica and Florisil (1 : 1, w/w, 8 g) using CH_2_Cl_2_ as eluent, to afford three major fractions for each species, which were then analyzed by GC-MS. Besides, the *n-*hexane fraction of *S. vinosa* (**SV-1**) was purified by column chromatography over silica gel 60 (0.063–0.200 mm, Merck) using *n*-hexane and EtOAc as eluents, which yielded compound **1** from fractions 32-33 (*n*-hexane/EtOAc 7 : 3 (v/v); 42 mg). On the other hand the EtOAc fraction of *S. vinosa* (**SV-2**) was purified by semipreparative reverse phase HPLC using MeOH/H_2_O/AcOH (45 : 54.9 : 0.1, v/v/v), UV detection at 254 nm, and flow rate 9 mL/min, furnishing compound **7** (7.9 mg). In a previous study, our research group had fractioned the crude extract of *M. langsdorffii* (**ML**, 6.7 g) and obtained six fractions as follows: **ML-1**: *n*-hexane/EtOAc 75 : 25 (v/v), **ML-2**: *n*-hexane/EtOAc 50 : 50 (v/v), **ML-3**: EtOAc, **ML-4**: AcOEt/EtOH 75 : 25 (v/v), **ML-5**: AcOEt/EtOH 50 : 50 (v/v), and **ML-6**: EtOH [15]. Fraction **ML-2** (500 mg) was the most active in the schistosomicial assay, and it was chromatographed over Celite and Norit (3 : 1, w/w, 60 g) using EtOAc as eluent. The resulting solid amorphous material was dissolved in MeOH/H_2_O 85 : 15 (v/v) and subsequently submitted to semi-preparative RP-HPLC purification using MeOH/H_2_O/AcOH (85 : 14.9 : 0.1, v/v/v), UV detection at 220 nm, and flow rate 9 mL/min, leading to compounds **2** (25 mg) and **3** (75 mg). The EtOAc fraction from *R. montana* (**RM-2**) followed to semi-preparative reverse phase HPLC purification, the analytical conditions were a mobile phase gradient consisting of MeOH/H_2_O/AcOH (47 : 52.9 : 0.1, v/v/v), UV detection at 254 nm, and a flow rate of 5 mL/min, yielding compound **4** (5.0 mg). Similarly, the *n*-BuOH fraction (**RM-3**) was submitted to semi-preparative reverse phase HPLC under the same conditions, providing compounds **5** (29.0 mg) and **6** (12.0 mg).

### 2.4. GC-MS Analysis

 A Shimadzu QP-2010 gas chromatograph equipped with HP-1 or DB-17MS capillary columns (30 m × 0.25 mm i.d. × 0.25 *μ*m film thickness) coupled to a mass spectrometer was employed. EI mass spectra were recorded at 70 eV. Conditions: for* S. vinosa n*-hexane fraction (**SV-1**): HP-1 column, injector 250°C; temperature program 100–290°C at 3°C/min, followed by 20-min isotherm; split ratio 1 : 30; carrier gas He at 1.10 mL/min flow rate. For* R. montana n*-hexane fraction (**RM-1**): DB-17MS column, injector 250°C; temperature program 100–290°C at 3°C/min, followed by 20-min isotherm; split ratio 1 : 20; carrier gas He at 1.10 mL/min flow rate. For* S. syringifolius n*-hexane fraction (**SS-1**): DB-17MS column, injector 250°C; temperature program 120–260°C at 3°C/min, followed by 5-min isotherm; then temperature program 260–280°C at 2°C/min, followed by 9-min isotherm; then temperature 280–290°C at 2°C/min, followed by 20-min isotherm; split ratio 1 : 50; carrier gas He at 1.40 mL/min flow rate. Identification of the constituents was conducted by computer search in the Wiley Mass Spectral Database 7.

### 2.5. *In Vitro* Schistosomicidal Assay

 The LE (Luis Evangelista) strain of *S. mansoni *was maintained by passage through *Biomphalaria glabrata *snails and BALB/c mice. After eight weeks, *S. mansoni* adult worms were recovered under aseptic conditions from mice previously infected with 200 cercariae by perfusion of the livers and mesenteric veins [21]. The worms were washed in Roswell Park Memorial Institute (RPMI) 1640 medium (Invitrogen), kept at pH 7.5 with HEPES 20 mM, and supplemented with penicillin (100 UI/mL), streptomycin (100 *μ*g/mL), and 10% bovine fetal serum (Gibco). After washing, two adult worms were transferred to each well of a 24-well culture plate containing 2 mL of the same medium and incubated at 37°C in a humid atmosphere containing 5% CO_2_ prior to use. At 24 h after incubation, extracts, fractions, and the isolated compounds (**1–7**) were dissolved in dimetilsulfoxide (DMSO) and added to RPMI 1640 medium, to give final concentrations of 50, 100, and 200 *μ*g/mL or *μ*M. The parasites were kept for 5 days and monitored each 24 h to evaluate their general condition. The worms were considered dead when no movement was observed for at least 2 min of examination and no movement at the other observation time points was detected [22]. Quadruplicate measurements were accomplished for each employed concentration and three independent experiments were performed. RPMI 1640 medium and RPMI 1640 with 1% DMSO (the highest concentration of drug solvent) were used as negative control groups. Praziquantel (PZQ) at 12.5 *μ*g/mL or 12.5 *μ*M was used as positive control group. All experiments were authorized by the Ethics Committee for Animal Care of the University of Franca and University of São Paulo, and they were in accordance with the national and international accepted principles for laboratory animal use and care.

## 3. Results and Discussion

**Figure 1:**
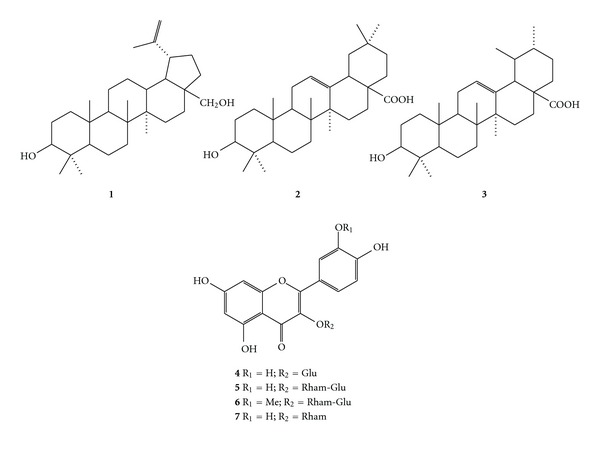
Chemical structures of the isolated compounds.

The scarcity of studies on the crude extract of *M. langsdorffii*, *R. montana*, *S. syringifolius*, and *S. vinosa* has encouraged us to accomplish biological and chemical investigations of metabolites belonging to these extracts. The chemical composition of the bioactive *n*-hexane fractions from EtOH or EtOH/H_2_O extracts of the aerial parts of *R. montana *(**RM-1**), *S. syringifolius* (**SS-1**), and *S. vinosa *(**SV-1**) was initially analyzed by GC-MS, and data are listed in Table 1. It can be noted that the identified compounds mainly belong to the following functional groups: aliphatic esters, hydrocarbons, steroids, and triterpenes. The presence of aliphatic esters is typical of all the investigated hexane fractions. **RM-1** contains the largest percent amount of these esters (8.89%), followed by **SV-1** (4.57%), but only trace amounts were detected in **SS-1** (0.30%). Triterpenes were identified in great quantities in **SS-1** (61.39%), followed by **SV-1** (29.24%), and **RM-1** (10.15%). Steroids were observed in **SV-1** (4.67%) and **SS-1** (4.91%). Hydrocarbons were detected in **SS-1** (19.39) and **RM-1** (8.03%). Compounds determined in minor quantities were sesquiterpenes, diterpenes, and tocopherol, which were only found in **SV-1** (7.84%), **RM-1** (17.91%), and **RM-1** (11.71%), respectively. The spectral profiles of the isolated compounds were in agreement with previously published data, which allowed identification of betulin (**1**) and quercetin 3-*O*-*β*-d-rhamnoside (**7**) in *S. vinosa *[23, 24]; oleanolic (**2**) and ursolic (**3**) acids in *M. langsdorffii* [23, 25] in addition to quercetin 3-*O*-*β*-d-glucoside (**4**), quercetin 3-*O*-*β*-d-glucopyranosyl-(1-2)-*α*-l-rhamnopyranoside (**5**) and isorhamnetin 3-*O*-*β*-d-glucopyranosyl-(1-2)-*α*-l-rhamnopyranoside (**6**) in *R. montana* (Figure 1) [24, 26]. To the best of our knowledge, this is the first report of the presence of compound **1** in *S. vinosa* and the occurrence of the flavonoids **4**, **5** and **6** in *R. montana*. In this study, the *in vitro* effect of the investigated extracts, fractions, and isolated compounds on *S. mansoni* parasite mortality was evaluated by incubation of the target microorganism with different concentrations and by evaluation of decrease in motor activity of this worm. In all the experiments, the negative control groups remained viable throughout the observation period. On the other hand, parasites belonging to the positive control group (PZQ) caused 100% parasite death on the first day of incubation. In addition no tegumental damage was observed in adult worms incubated with the evaluated crude extracts, fractions, and isolated compounds. The tegument is extremely important for parasite survival and infection success within the host, and it has been a major target for the development of drugs against *Schistosoma* [20, 22]. Except for **SS**, all the studied crude extracts displayed some effect on *S. mansoni* mortality (Table 2). On the first day of incubation, crude extract **ML** at a concentration of 100 *μ*g/mL caused 25% adult worms mortality. In addition, on the fifth day of incubation 100% parasite mortality was achieved with extract **ML** at concentration of 100 *μ*g/mL and also with extracts **RM** and **SV** at concentrations of 200 *μ*g/mL. On the other hand extracts **SS** and **SV** displayed significant reduction in motor activity at 50, 100 and 200 *μ*g/mL. The occurrence of lethal effect on the first treatment day was noted for fractions **RM-2**, **SS-1**, **SV-2**, and **ML-2** at a concentration of 100 *μ*g/mL; however, on the fifth treatment day, fraction **RM-2** prompted 100% mortality at concentrations of 50 *μ*g/mL and 100 *μ*g/mL and fractions **SV-1** and **SV-2** caused 100% parasite mortality at concentrations of 100 *μ*g/mL and 200 *μ*g/mL. Furthermore fractions **RM-1**, **SS-1**, **SV-1**, and **ML-2** promoted considerable reduction in motor activity at the assayed concentrations.

Fractions **SV-1**and **ML-2 ** fractions were selected for a further purification process, in which they were chromatographed over silica using an *n*-hexane/EtOAc gradient. **SV-1** purification readily furnished **1**. Compounds **2** and **3** were isolated after semi-preparative HPLC of **ML-2 **[15]. As observed in Table 3, the isolated compounds **2** and **3** did not have lethal effects on *S. mansoni* adult worms, thus showing loss of activity during the phytochemical procedures. On the other hand, betulin (**1**) at a concentration of 200 *μ*M caused 50% parasite mortality on the fifth day of incubation. In addition, 25% mortality was verified on the fifth treatment day at a concentration of 100 *μ*M and demonstrated 50% of reduction in motor activity within 24 h. Thus, betulin (**1**) has also been demonstrated to exert an effect on *S. mansoni* adult worm mortality. It is noteworthy that the structures of compounds **1**, **2**, and **3** are quite similar, differing mainly in the presence of a five-membered ring and an alcohol moiety in **1** as compared to the existence of a six-membered ring and an acid group in **2** and **3**. Therefore, bearing in mind parasite viability, it is suggested that the presence of the five-membered ring and the alcohol functional group in **1** may improve the activity of triterpenes derivatives against *S. mansoni*, since among compounds **1**, **2**, and **3** only compound **1** caused parasite death. However, the action of betulin *in vitro* against chloroquine resistant (K1) and sensitive (T9–96) *Plasmodium falciparum* strains has already been assessed, and it was found to be inactive [27]. On the other hand the semi-preparative RP-HPLC study of fractions **RM-2**, **RM-3**, and **SV-2** afforded the flavonoids **4**–**7**. Considering the schistosomicidal activity results of the flavonoids isolated summarized in Table 3, we can observe that only quercetin 3-*O*-*β*-d-rhamnoside (**7**), also known as quercitrin, was able to cause 25% parasite death on the fifth day of treatment at a concentration of 100 *μ*g/mL. Concerning the reduction of motor activity of the parasites compared with the negative control, the flavonoid monoglycosides **4** and **7** at 100 *μ*M exhibited significantly reduction in motor activity of 75% and 87.5%, respectively. However, flavonoids **5** and **6** were inactive. Our results related the isolated flavonoids assayed suggest that the monoglycosylation at C-3 position in ring C increases the reduction in motor activity in *S. mansoni* adult worm. Previous investigations on the schistosomicidal activity of natural products realized by our research group reveal that aglycone quercetin was not able to kill the worms but exhibited moderately reduced motor activity [28]. However, quercetin was identified as a selective inhibitor of the *S. mansoni* NAD^+^ catabolizing enzyme (*Sm*NACE), localized to the outer surface (tegument) of the adult parasite and presumably involved in the parasite survival by manipulating the host's immune regulatory pathways. These studies identified that the nature of ring C and the substitution of free hydroxyl groups in rings A, B, and C in flavonoids are key structural features for SmNACE inhibition [29].

The mechanism by which the extracts, fractions, and betulin (**1**) and flavonoids **4** and **7** exert their effects remains unclear. Moreover, as suggested by our results, the lupane-type triterpene and flavonoid monoglycosides should also be considered for further antiprotozoal studies. In summary, chemical investigations of metabolites from the selected species resulted in the isolation and identification of compounds **1**–**7**. Additionally, biological results indicated that crude extracts **RM**,** SV**, and **ML**; fractions **RM-1**, **RM-2**, **SS-1**, **SV-1**, **SV-2**, and **ML-2**; the triterpene betulin (**1**); the flavonoids quercetin 3-*O*-*β*-d-glucoside (**4**); quercetin 3-*O*-*β*-d-rhamnoside (**7**) are able to induce worm death to some extent as well as to reduce the motor activity of the parasites. Additional chemical studies are in progress in our research group to identify other natural compounds related to schistosomicidal action of the species investigated. The knowledge of chemical composition and schistosomicidal potential of *R. montana*, *S. syringifolius*, *S. vinosa*, and *M. langsdorffii* will provide insight information for the future application of these plants.

## Figures and Tables

**Table 1:** CG/MS identification of the main chemical constituents of the *n*-hexane fractions of *R. montana* (**RM-1**),* S. syringifolius *(**SS-1**), and *S. vinosa* (**SV-1**).

Source/compound	M^+^	*t*_*R*_ (min)	Concentration (%)
*R. montana *(**RM-1**)			
Ethyl pentadecanoate	270	29.935	8.89
Fytol	296	33.653	17.91
Squalene	410	52.799	4.18
*γ*-tocopherol	416	60.342	11.71
Lupeol	426	72.546	8.05
*β*-amyrin	426	70.015	1.34
*α*-amyrin	426	72.230	0.76
*n*-tetracontane	619	51.188	3.85
*S. syringifolius *(**SS-1**)			
Methyl palmitate	270	5.842	0.30
*n*-heneicosane	296	9.437	2.74
*n*-nonacosane	408	11.562	13.57
Clionasterol	414	27.655	1.21
Lupeol	426	34.065	22.03
*β*-amyrin	426	31.128	12.75
*α*-amyrin	426	33.706	5.04
9,19-ciclolanostan-24-en-3-ol	426	31.883	1.74
*β*-friedelanol	428	38.254	10.29
9,19-ciclolanostan-3-ol, 24-methylene	440	33.456	1.96
*α*-amyrin acetate	468	35.235	1.41
Lupeol acetate	468	35.680	9.87
*n*-tetracontane	619	14.624	3.08
*S. vinosa *(**SV-1**)			
Spathulenol	220	18.130	7.84
Ethyl palmitate	284	32.450	4.57
Stigmasterol	412	63.660	3.28
Sitosterol	414	65.045	1.39
Lupenone	424	66.225	1.33
Lupeol	426	67.015	27.91

**Table 2:** *In vitro* effects of the crude extract and fractions against *S. mansoni* adult worms.

Group	Incubation period (h)	% of dead worms at a given			% reduction in motor activity		
		concentration (*μ*g/mL)			at a given concentration (*μ*g/mL)		
		50	100	200	50	100	200
**ML**	24	0	25	n.t.	0	75	n.t.
	120	0	100	n.t.	0	0	n.t.
**ML-2**	24	0	37.5	n.t.	12.5	0	n.t.
	120	50	62.5	n.t.	37.5	12.5	n.t.
**RM**	24	0	0	0	0	0	25
	120	0	0	100	0	0	0
**RM-1**	24	0	0	0	75	75	25
	120	0	100	100	100	0	0
**RM-2**	24	0	25	n.t.^a^	0	75	0
	120	100	100	n.t.	0	0	0
**RM-3**	24	0	0	0	0	0	0
	120	0	0	0	0	0	0
**SS**	24	0	0	0	25	100	100
	120	0	0	0	50	100	100
**SS-1**	24	0	25	25	100	75	75
	120	25	25	25	75	75	50
**SS-2**	24	0	0	0	0	0	25
	120	0	0	0	0	0	25
**SS-3**	24	0	0	0	0	0	25
	120	0	0	0	0	25	25
**SV**	24	0	0	0	0	75	100
	120	0	25	100	100	100	0
**SV-1**	24	0	0	100	25	50	0
	120	0	100	100	100	0	0
**SV-2**	24	0	25	100	0	25	0
	120	0	100	100	0	0	0
**SV-3**	24	0	0	0	0	0	0
	120	0	0	100	0	0	0
Control^b^	24	0	0	0	0	0	0
	120	0	0	0	0	0	0
1% DMSO	24	0	0	0	0	0	0
	120	0	0	0	0	0	0

^a^n.t.: not tested.

^b^RPMI 1640.

PZQ at 12.5 *μ*g/mL  = 100% parasite death on 24 h of incubation.

**Table 3:** *In vitro* effects of isolated compounds against *S. mansoni* adult worms.

Group	Incubation period (h)	% of dead worms at a given			% reduction in motor activity		
		concentration (*μ*M)			at a given concentration (*μ*M)		
		50	100	200	50	100	200
**1**	24	0	0	25	0	50	25
	120	0	25	50	0	50	0
**2**	24	0	0	0	0	0	25
	120	0	0	0	0	0	25
**3**	24	0	0	0	25	0	50
	120	0	0	0	25	25	50
**4**	24	0	0	n.t.^a^	50	75	n.t.
	120	0	0	n.t.	50	75	n.t.
**5**	24	0	0	n.t.	0	0	n.t.
	120	0	0	n.t.	0	0	n.t.
**6**	24	0	0	n.t.	0	25	n.t.
	120	0	0	n.t.	0	0	n.t.
**7**	24	0	0	n.t.	75	87.5	n.t.
	120	0	25	n.t.	75	87.5	n.t.
Control^b^	24	0	0	0	0	0	0
	120	0	0	0	0	0	0
1% DMSO	24	0	0	0	0	0	0
	120	0	0	0	0	0	0

^a^n.t.: not tested.

^b^RPMI 1640.

PZQ at 12.5 *μ*M  = 100% parasite death on 24 h of incubation.
